# Effect of Aging and Dietary Salt and Potassium Intake on Endothelial PTEN (Phosphatase and Tensin Homolog on Chromosome 10) Function

**DOI:** 10.1371/journal.pone.0048715

**Published:** 2012-11-07

**Authors:** Wei-Zhong Ying, Kristal J. Aaron, Paul W. Sanders

**Affiliations:** 1 Division of Nephrology, Department of Medicine, Nephrology Research and Training Center, Center for Free Radical Biology, Center for Aging, and Department of Cell, Developmental and Integrative Biology, University of Alabama at Birmingham, Birmingham, Alabama, United States of America; 2 Department of Veterans Affairs Medical Center, Birmingham, Alabama, United States of America; Indiana University, United States of America

## Abstract

Aging promotes endothelial dysfunction, defined as a reduction in bioavailable nitric oxide (NO) produced by the endothelial isoform of nitric oxide synthase (NOS3). This enzyme is critically regulated by phosphorylation by protein kinase B (Akt), which in turn is regulated by the lipid phosphatase, PTEN. The present series of studies demonstrated a reduction in bioavailable NO as the age of rats increased from 1 to 12 months. At 12 months of age, rats no longer demonstrated increases in phosphorylated NOS3 in response to high dietary salt intake. Endothelial cell levels of PTEN increased with age and became refractory to change with increased salt intake. In contrast to the reduction in NO production, endothelial cell production of transforming growth factor-ß (TGF-ß) relative to NO increased progressively with age. In macrovascular endothelial cells, PTEN was regulated in a dose-dependent fashion by TGF-ß, which was further regulated by extracellular [KCl]. When combined with prior studies, the present series of experiments suggested an integral role for PTEN in endothelial cell pathobiology of aging and an important mitigating function of TGF-ß in endothelial PTEN regulation. The findings further supported a role for diet in affecting vascular function through the production of TGF-ß and NO.

## Introduction

In 1980, Furchgott published his seminal report that demonstrated the integral function of the endothelium in vasomotor tone [1]. Remarkable ongoing effort has since characterized the role of nitric oxide (NO) in endothelium-dependent vasodilation and further identified the association of endothelial dysfunction of the macro- and microcirculation, manifested as a reduction in bioavailable endothelium-derived NO, with the development of cardiovascular disease [2], [3], a leading cause of death in the United States [4]. While disease states such as diabetes mellitus, hypertension, and atherosclerosis are related to impaired endothelial function [2], [3], [5], [6], endothelial dysfunction is also a consequence of the aging process [7]–[11].

Vascular pathology associated with endothelial dysfunction of aging involves not only resistance vessels, but also includes remodeling and subsequent stiffening of compliance vessels [12], [13]. In a study of late-middle-aged non-smokers who did not have clinical cardiovascular disease or diabetes mellitus, decreased systemic flow-mediated dilation predicted progressive thickening of the carotid artery [14]. It is now apparent that the disease burden related to conduit arterial pathology and stiffness is very high. In the Cardiovascular Health Study, a prospective multicenter study of 4,476 men and women 65 years of age or older, 3,579 (79.9%) had increased carotid artery intima-media thickness. While these individuals did not have a history of cardiovascular disease at enrollment, increasing thickness of the carotid artery was directly associated with subsequent progressive risk of myocardial infarction and stroke [15]. Arterial stiffness may also stimulate left ventricular hypertrophy [12], [16] and remodeling of the microcirculation particularly in the brain and kidney, two organs subjected to high perfusion flow rates and pulsatile pressures [17], [18]. As an independent predictor of cardiovascular/renal morbidity and mortality, conduit artery stiffness associated with endothelial dysfunction therefore represents an important health problem.

Mice lacking the endothelial isoform of nitric oxide synthase (NOS3) develop increased aortic stiffness compared to wild-type mice [13], indicating the critical role that bioavailable NO plays in the remodeling of compliance vessels. This enzyme normally produces NO throughout the vasculature and is regulated in a complex fashion. Not only is calcium/calmodulin activation of NOS3 responsible for transient increases in NO, but post-translational phosphorylation events provide more prolonged NO release by NOS3 at baseline and following stimulation [19]–[21]. In particular, NOS3 serves as a substrate for protein kinase B (Akt) [19], which promotes serine phosphorylation at residue 1179 in the autoinhibitory carboxyl terminal portion of bovine NOS3 and thereby increases NOS3 sensitivity to calcium/calmodulin and enzyme activity [22]. This latter regulatory mechanism is especially important in the setting of changes in dietary salt intake [23].

**Table 1:** Summary of physiological parameters of the groups of rat under study.

Age(months)	DietaryNaCl (%)	BW (g)	MAP(mm Hg)	SerumCreatinine(mg/dl)	Urinary NOx(nmol/h per 100 g BW)	Urinary ActiveTGF-ß(fmol/h per 100 g BW)
1	0.3	166±3	91±3	0.10±0.01	14.5±2.5	2.7±0.6
1	8.0	154±5	97±3	0.11±0.00	32.6±4.0	34.1±3.9
6	0.3	401±7	108±1	0.22±0.03	6.4±0.4	1.8±0.7
6	8.0	400±2	105±5	0.18±0.02	8.5±1.0	9.0±1.2
12	0.3	463±13	123±6	0.22±0.01	4.6±0.4	2.5±0.5
12	8.0	437±12	114±9	0.20±0.01	7.6±0.8	12.3±3.2

See text for details.

PTEN (Phosphatase and tensin homologue deleted on chromosome 10) is a lipid phosphatase that was discovered 25 years ago [24]. PTEN specifically serves as a 3-phosphatase for phosphatidylinositol 3,4,5-triphosphate (PIP3) [25] and is a critically important regulator of this important signaling molecule that influences fundamental cellular activities [26], [27]. PTEN antagonizes the 3-kinase activity of the class I phosphatidylinositol 3-kinase (PI3K) family, which generates PIP3 following activation, and levels of PTEN appear to determine cellular levels of PIP3 [26]–[29]. Among other effects, increased PIP3 levels promote Akt activation [30]. Thus, endothelial PTEN might regulate NO production through Akt. The hypothesis tested in this paper is that production of bioavailable NO declines with age and in response to dietary salt supplementation and is related to impaired Akt-mediated phosphorylation of NOS3. The integral involvement of PTEN in this aging process was determined.

**Figure 1:**
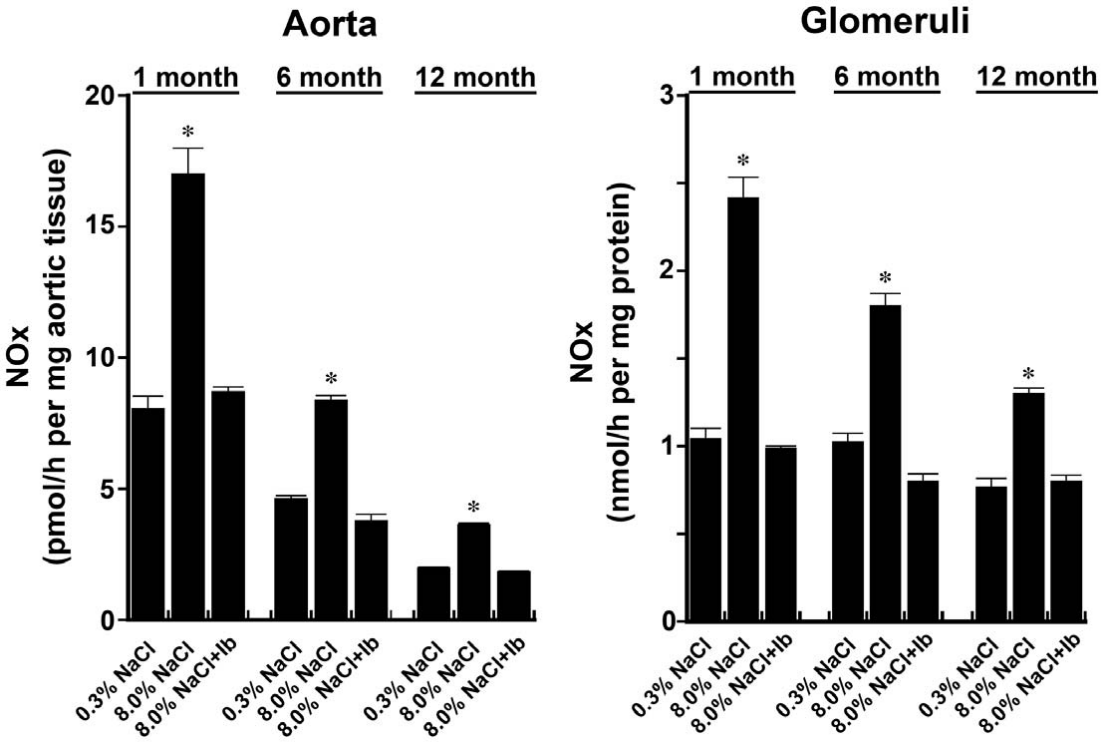
Production of bioavailable NO by aortic ring segments and isolated glomeruli from rats age 1, 6, and 12 months. NOx production rates by tissue from rats that received 8.0% NaCl diet were greater than tissues from rats that were the same age and on the 0.3% NaCl diet. Addition of iberiotoxin (IB, 100 nM) inhibited the salt-induced production of NOx in all three groups. *p-value<0.05 compared to the other groups of rats the same age. n = 4−6 rats in each group.

**Figure 2:**
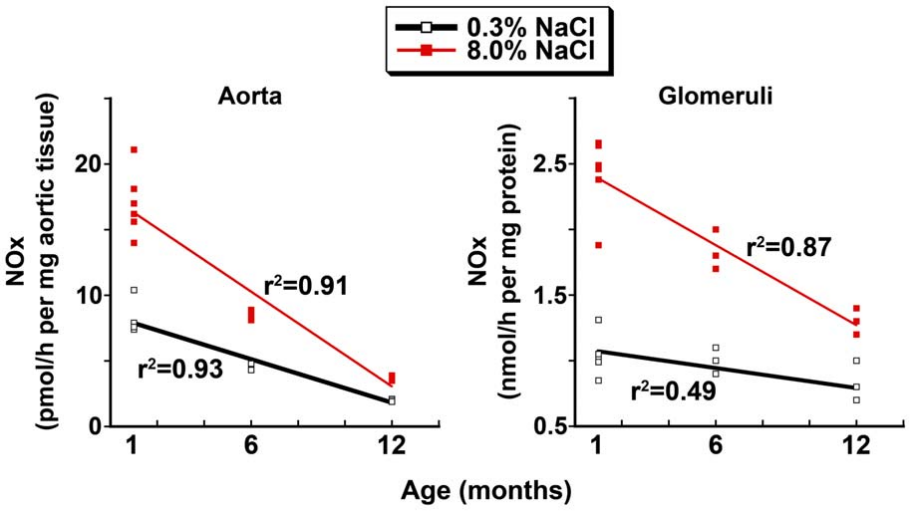
NOx production rates by vascular tissues (aorta on left and isolated glomeruli on right) obtained from rats 1, 6 and 12 months of age. Findings from animals on 8.0% and 0.3% NaCl diets were analyzed separately. For aortic rings and isolated glomeruli from rats on both diets, a statistically significant inverse linear correlation was observed (p-values<0.05), indicating a steady decline in bioavailable NO in these tissue with age. Each square in the graphs represents data from a single rat.

**Figure 3:**
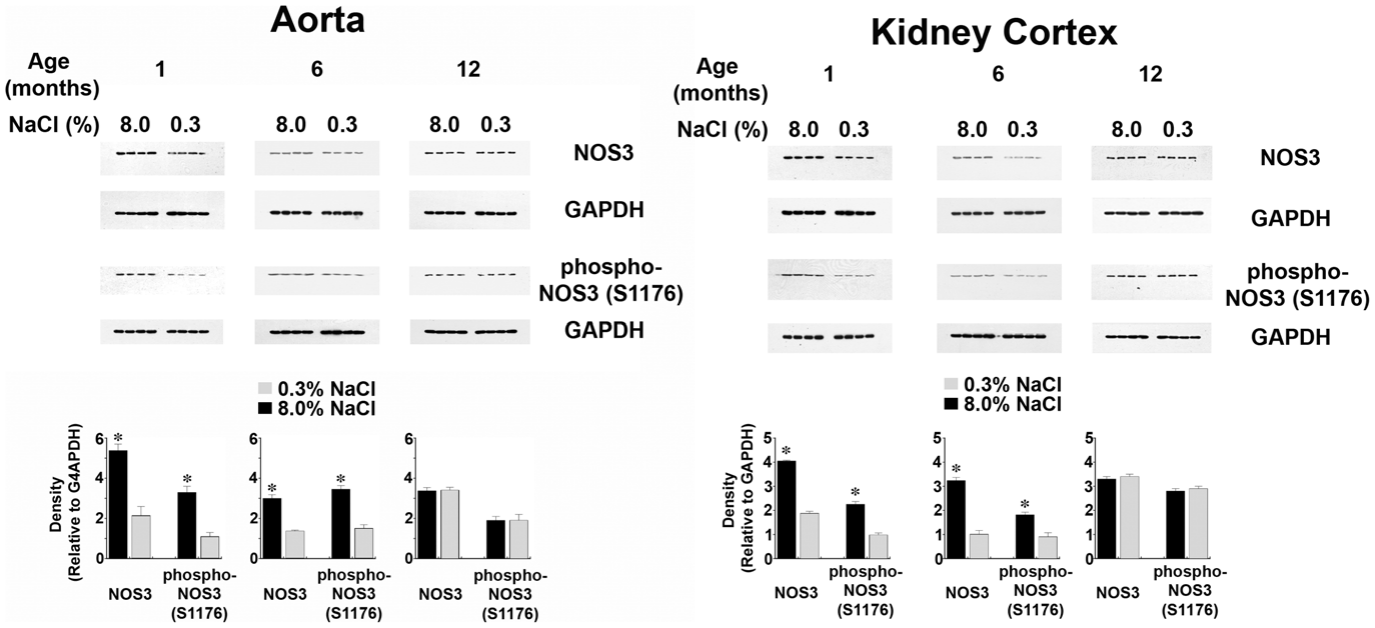
Effect of aging on NOS3 and phospho-NOS3(S1176) levels in aorta and kidney cortex. Tissues from rats age 1 and 6 months, but not tissues from the 12-month old rats, demonstrated an increase in NOS3 and phospho-NOS3(S1176) levels in response to the high-salt diet. n = 4 rats in each group. *p-value<0.05 compared to the corresponding group of rats maintained on 0.3% NaCl diet for 4 days.

## Materials and Methods

### Animal and Tissue Preparation

This study was carried out in strict accordance with the recommendations in the Guide for the Care and Use of Laboratory Animals of the National Institutes of Health. The Institutional Animal Care and Use Committee at the University of Alabama at Birmingham approved the project. Studies were conducted using 48 male Sprague-Dawley rats (Harlan Laboratories, Indianapolis, IN) ages 1, 6, and 12 months. The rats were housed under standard conditions and given 0.3% NaCl diet (AIN-76A with 0.3% NaCl; Dyets, Inc., Bethlehem, PA) and water ad libitum for 4 days before initiating the experiment. At the start of the experiment, the rats were placed in metabolic cages and allowed free access to water and diet, which contained either 0.3% or 8.0% (AIN-76A with 8.0% NaCl; Dyets, Inc., Bethlehem, PA) NaCl. These diets were prepared specifically to be identical in protein composition and differed only in NaCl and sucrose content. Urine was collected under oil to prevent desiccation. Food consumption, urine flow rates, and body weight were monitored daily. Blood pressure (BP) was determined noninvasively using a blood pressure analysis system (Model SC1000, Hatteras Instrument, Inc., Cary, North Carolina). Each rat was subjected daily to ∼15 measurements, of which flawed readings from movement- or machine-related issues, for example, were omitted. Blood pressures from the measurements on each day were averaged for each rat. The experiment was concluded after the 4^th^ day. Collected urine samples were filtered to remove any particulate matter and centrifuged at 325×g for 2 min at 4°C. The supernatant was immediately frozen at −80°C until use.

On the final day of study, rats were anesthetized with pentobarbital sodium (Nembutal, Lundbeck Inc., Deerfield, IL), 50 mg/kg body weight, i.p. Blood was collected for determination of serum creatinine, which was assayed using liquid chromatography tandem mass spectrometry (Waters 2795 LC-MS/MS, Waters Corporation, Milford, MA) [31]. Aorta and both kidneys were harvested under sterile conditions for isolation of glomeruli for in vitro incubation experiments or to generate tissue lysates for protein analyses. Aortic endothelial cell lysates were obtained as described previously [32].

### In vitro Incubation Studies

After removal of adherent fat and connective tissue, the aortic tissue was rinsed in ice-cold PBS and cut into 3-mm ring segments. Glomeruli were isolated using a graded sieving technique. This protocol has been shown to produce >95% pure and viable glomeruli [33]. Pelleted glomeruli were washed and resuspended at 5×10^3^ glomeruli/ml of serum-free medium (DMEM; Life Technologies, Grand Island, NY). In some experiments, tissue samples were incubated in medium that contained vehicle alone or 100 nM iberiotoxin (Ib; Sigma-Aldrich, Inc., St. Louis, MO). After incubation for 24 h, the conditioned medium was harvested, centrifuged at 300×g for 10 min at 4°C to remove cell debris, and then stored at −80°C until assayed for NOx and total and active TGF-β.

**Figure 4:**
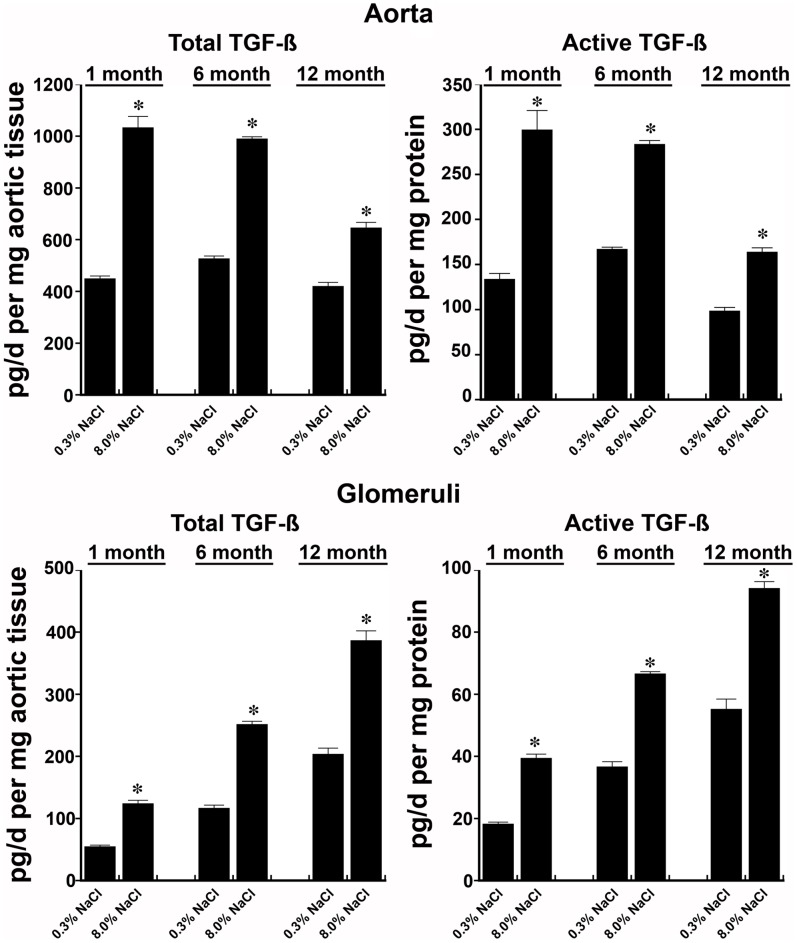
TGF-ß production rates by aortic rings and isolated glomeruli from rats age 1, 6, and 12 months. Total and active TGF-ß production rates by tissue from rats that received 8.0% NaCl diet were greater than tissues from rats that were the same age and on the 0.3% NaCl diet. *p-value<0.05 compared to the other groups of rats the same age. n = 4−6 rats in each group.

**Figure 5:**
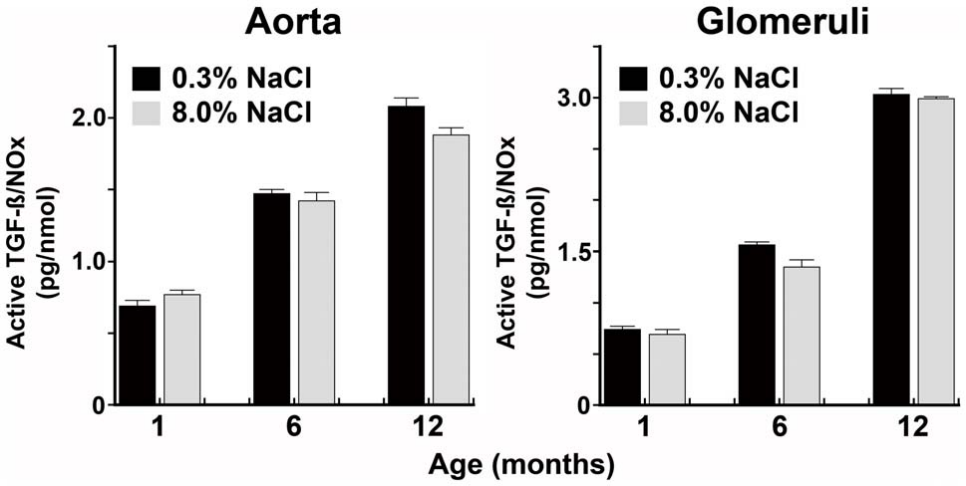
The production of active TGF-ß relative to NOx progressively increases with age. Least square means comparison demonstrated a strongly significant linear effect for increasing active TGFß/NO (pg/nmol) with age for both aortic rings and isolated glomeruli (all p-values<0.0001).

### Human Umbilical Vein Endothelial Cells (HUVEC) Incubation Studies

Primary cultures of macrovascular endothelial cells (HUVEC) were obtained commercially (Life Technologies, Grand Island, NY). Monolayers of HUVEC were incubated at 37°C with 5% CO_2_/95% air in Medium 200 (Life Technologies, Grand Island, NY). Medium was exchanged at 48-h intervals and cells were not used beyond 25–30 passages.

At the initiation of study, monolayers of HUVEC in 6-well plates were incubated in Medium 200 that was produced without potassium by the manufacturer. During these experiments, the medium was also supplemented with Low Serum Growth Supplement (LSGS, Life Technologies, Grand Island, NY), which resulted in a final concentration of 2% (v/v) fetal bovine serum. This medium permitted addition of KCl to final concentrations between 0 and 5 mEq/L; choline chloride (Sigma-Aldrich, Inc., St. Louis, MO), 0–5 mEq/L, was added to maintain constant osmolality among the groups. The plates were incubated for 24 h at 37°C before study. In other experiments, HUVEC were incubated in Medium 200 containing 0 or 5 mEq/L of KCl; Medium 200 with 0 KCl and 5 mEq/L of NaCl served as a control in these studies. Some experiments also included the addition of iberiotoxin (Sigma-Aldrich, Inc., St. Louis, MO), 100 nM, vehicle, or glibenclamide (Sigma-Aldrich, Inc., St. Louis, MO), 10 µM. Iberiotoxin served as a selective and reversible inhibitor of high-conductance calcium-activated potassium channels (BK_Ca_) [34] and glibenclamide was a potent inhibitor of ATP-dependent potassium channels [35]. In other experiments, monolayers of HUVEC were incubated in 75-cm^2^ flasks with Medium 200 containing 0 or 5 mEq/L of potassium and either 10 µg/ml rabbit polyclonal antibody that specifically neutralizes TGF-ß (R&D Systems, Minneapolis, MN) or 10 µg/ml of nonspecific rabbit IgG (Southern Biotechnology Associates, Birmingham, AL), which served as a control. After 24-h incubation, the conditioned medium was harvested, centrifuged at 300×g for 10 min at 4°C to remove cell debris, and then stored at −80°C until assayed for total and active TGF-ß. Cell lysates were obtained for analysis of PIP3, PTEN, Akt, NOS3, GAPDH, and total protein concentration.

**Figure 6:**
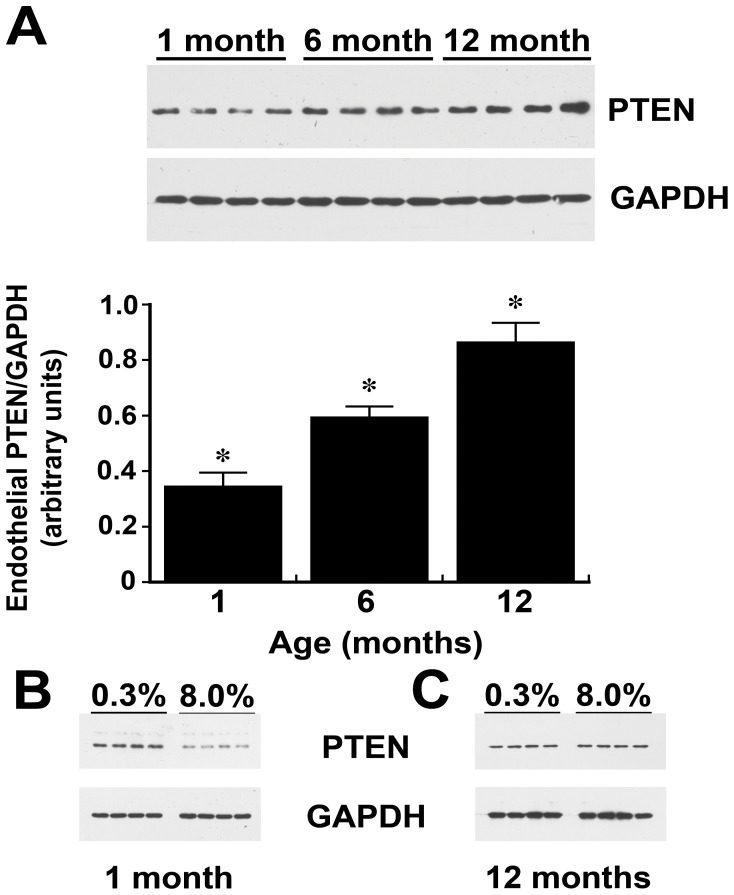
Endothelial PTEN levels increase with age and become refractory to change. (A) Western blot showing increases in endothelial PTEN levels with aging. The graph shows the mean density of the bands relative to GAPDH. n = 4 rats in each group. *p-value <0.05 compared to the other two groups (B) Endothelial PTEN levels of one-month old rats on 0.3% and 8.0% NaCl diets. Endothelium from rats on the low-salt diet had greater amounts of PTEN relative to GAPDH than endothelium from rats the same age on the high-salt diet (0.56±0.03 versus 0.17±0.01; p-value <0.05). (C) Endothelial PTEN levels of 12-month old rats on 0.3% and 8.0% NaCl diets. In this analysis, endothelium from rats on the low-salt and high-salt diets had comparable amounts of PTEN relative to GAPDH (0.072±0.002 versus 0.074±0.001; p-value = 0.422).

**Figure 7:**
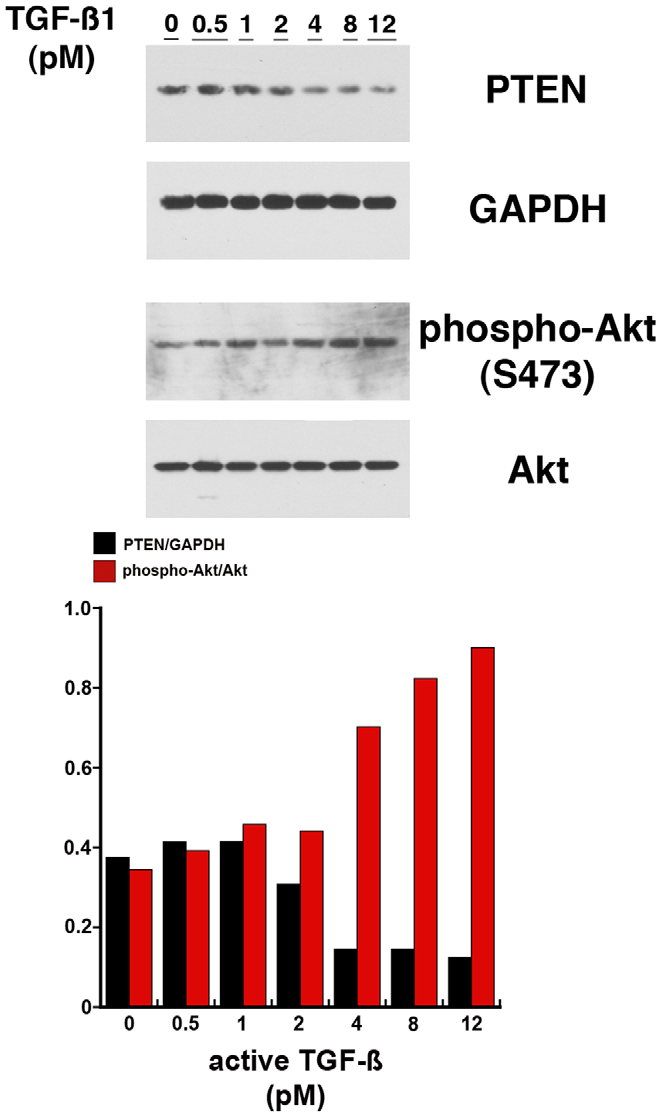
Macrovascular endothelial cells (HUVEC) incubated overnight in medium supplemented with increasing amounts of active TGF-ß1 demonstrate a dose-dependent reduction in PTEN and a corresponding increase in phospho-Akt(S473). Error bars were not included as this experiment was performed once.

### TGF-ß Assay

Determination of concentrations of total and active TGF-ß was performed as described [32]. To determine urinary active TGF-β, urine samples were diluted 1∶5 in a total volume of 1 ml of serum-free medium, with pH adjusted with 0.1 N of NaOH, and added to wells of 24-well plates. For assay of total TGF-β, urine samples were heated for 5 min at 100°C, diluted 1∶20 in a total volume of 1 ml of serum-free medium, and incubated with reporter cells for 18 h at 37°C. Mink lung epithelial cell lysates from each well were prepared using reporter lysis buffer (Promega Corporation, Madison, WI). Luciferase activity was determined as relative light units using a microplate luminometer (Clarity Luminescence Microplate Reader; BioTek Instruments, Winooski, VT) and converted to concentration of TGF-β using a standard curve generated using human recombinant TGF-β1 (R&D Systems, Minneapolis, MN). To determine TGF-ß in medium, 200 µl of medium were diluted 1∶3 in serum-free medium (DMEM; Life Technologies, Grand Island, NY) with 0.1% BSA before adding to the wells of 24-well plates. The results were factored by wet weight (for aortic tissue) or total protein (for glomeruli).

### Nitric Oxide Metabolites

Nitric oxide metabolites (NOx) in samples of urine and medium were assayed using an optimized Cd/Cu reagent kit (QuantiChrom Nitric Oxide Assay Kit, BioAssay Systems, Hayward, CA), which determines nitrite concentrations using Griess methodology following reduction of nitrate to nitrite. In these studies, assays were performed in triplicate and averaged. Results using tissue samples were factored by wet weight (for aortic tissue) or total protein (for glomeruli).

### Determination of Phosphatidylinositol 3,4,5-triphosphate (PIP3)

The amount of cellular PIP3 was determined using an ELISA (PIP_3_ Mass ELISA Kit™, Echelon Biosciences Inc., Salt Lake City, UT) and is based upon a novel approach that uses a labeled protein that contains a pleckstrin homology [36]. This assay is a competitive 96-well ELISA, in which the signal is inversely proportional to the amount of PIP3 in the sample. PIP3 was extracted from pelleted HUVEC according to the protocol provided by the manufacturer. In these studies, assays were performed in duplicate and averaged.

**Figure 8:**
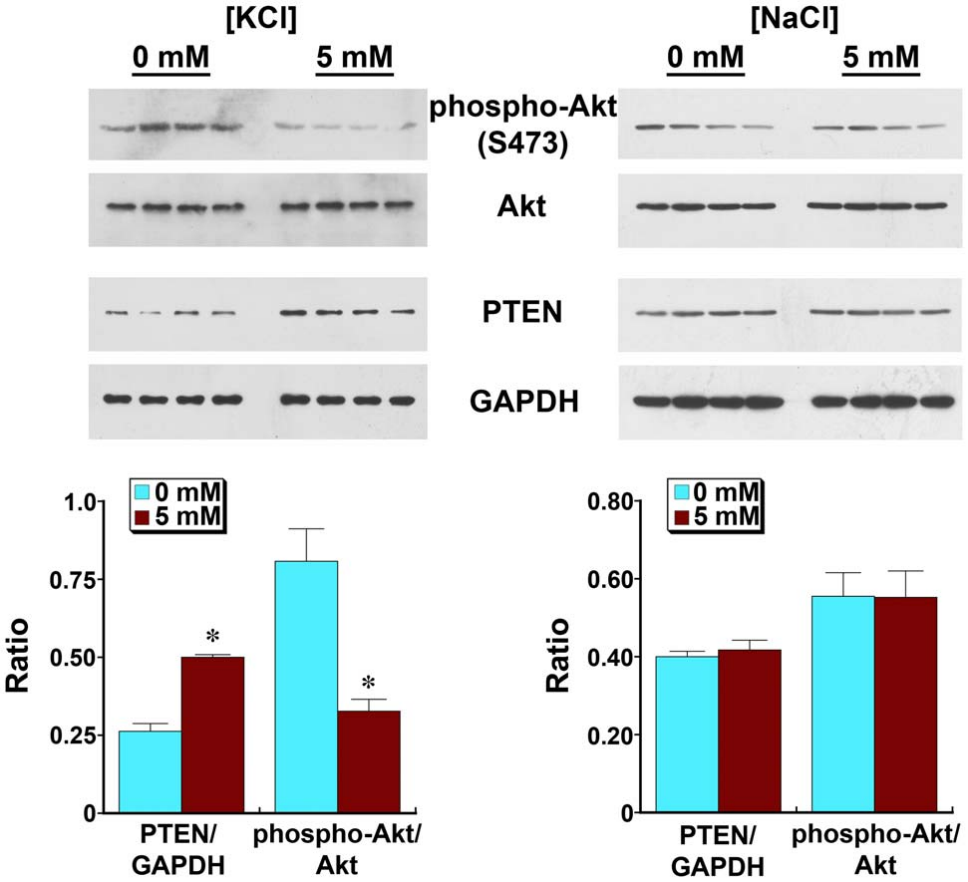
Ambient potassium chloride, but not sodium chloride, regulates PTEN levels and Akt function in endothelial cells. Macrovascular endothelial cells (HUVEC) were incubated overnight in medium containing [KCl] at 0 and 5 mM (left panel) or in medium containing [KCl], 0 mM, supplemented with 0 or 5 mM [NaCl] (right panel). Compared to cells incubated in medium lacking KCl, cells incubated in medium containing 5 mM KCl demonstrated less phospho-Akt(S473) and more PTEN, relative to GAPDH. In contrast, overnight incubation in medium supplemented with NaCl instead of KCl did not alter intracellular PTEN or phospho-Akt(S473). Each lane represents data from a single experiment.

**Figure 9:**
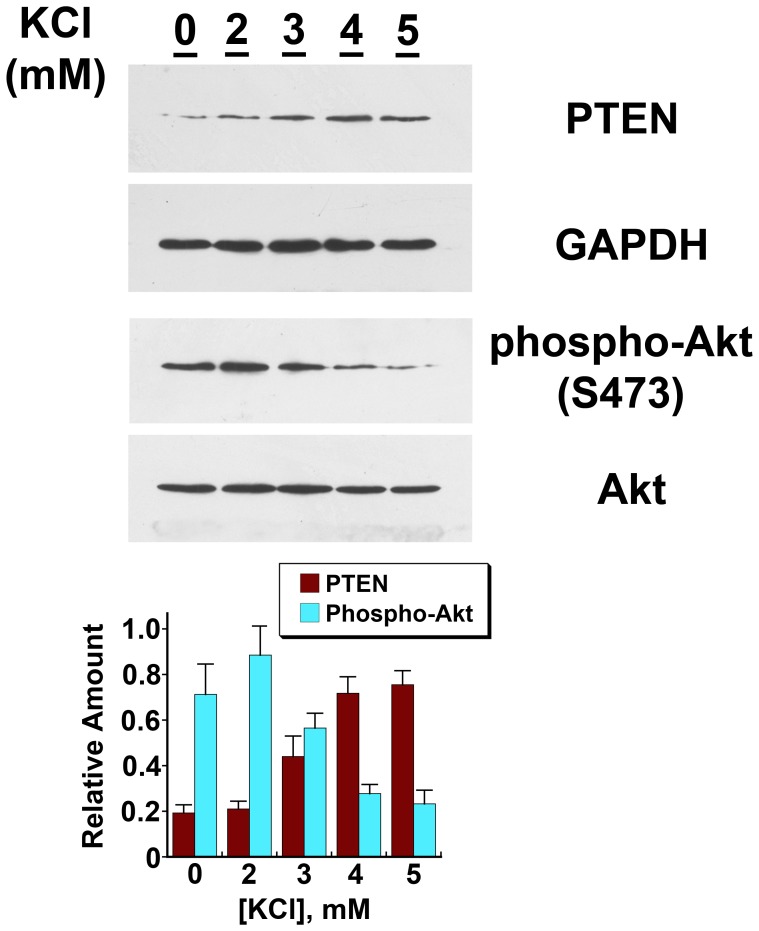
Potassium chloride regulates PTEN levels and Akt function in endothelial cells in a dose-dependent fashion. The western blot shows the results of a single experiment, while the graph shows the data from a total of four separate experiments. Incubating HUVEC overnight in medium containing increasing amounts of [KCl] resulted in a progressive statistically significant (p-value <0.05) increase in PTEN levels and an associated decline in phospho-Akt(S473). For both PTEN and phospho-Akt(S473), data from cells incubated in [KCl], 0 and 2 mM, differed (p-value <0.05) from data derived from cells incubated in [KCl], 4 and 5 mM.

**Figure 10:**
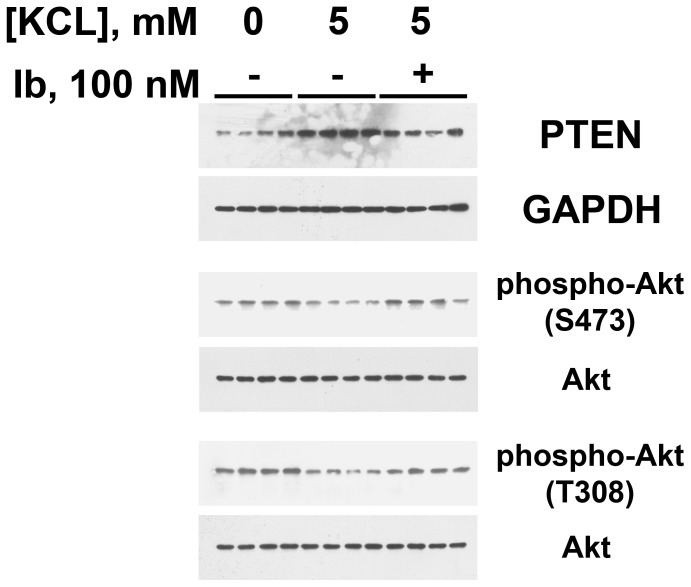
The effect of ambient [KCl] on endothelial PTEN and phosphorylation of Akt at S473 and T308 was dependent upon BK_Ca_ activity. In these experiments, each lane represents a single experiment. Overnight incubation of HUVEC in medium containing [KCl], 5 mM, and iberiotoxin (Ib), 100 nM, reduced PTEN levels (top panel) to those observed in cells incubated in medium containing 0 mM [KCl] (PTEN/GAPDH ratios for [KCl] 0, [KCl] 5 and [KCl] 5 plus Ib were 0.53±0.07, 0.99±0.05, and 0.61±0.07; p-value <0.05). Incubation of HUVEC in medium containing [KCl], 5 mM, reduced phospho-Akt(S473) (middle panel), compared to values obtained from HUVEC incubated in medium without KCl; this decrease was reversed by the addition of Ib (phospho-Akt(S473)/Akt ratios for [KCl] 0, [KCl] 5 and [KCl] 5 plus Ib were 0.50±0.04, 0.17±0.03, and 0.48±0.05; p-value <0.05). Incubation of HUVEC in medium containing [KCl], 5 mM, reduced phospho-Akt(T308) (bottom panel), compared to values obtained from HUVEC incubated in medium without KCl; addition of Ib increased phospho-Akt(T308)/Akt ratios. For [KCl] 0, [KCl] 5 and [KCl] 5 plus Ib, the ratios were 0.65±0.02, 0.23±0.02, and 0.46±0.02; p-value <0.05.

### Western Blot Analyses

Tissue and cell lysates were produced using modified radioimmunoprecipitation assay (RIPA) buffer that contained 10 mM Tris·HCl, pH 7.4, 100 mm NaCl, 1 mM EDTA, 1 mM EGTA, 0.5% sodium deoxycholate, 1% Triton X-100, 10% glycerol, 0.1% SDS, 20 mM sodium pyrophosphate, 2 mM Na_3_VO_4_, 1 mM NaF, 1 mM PMSF, and a protease inhibitor cocktail. Total protein concentration was determined using a kit (BCA Protein Assay Reagent Kit; Thermo Fisher Scientific Pierce Protein Research Products, Rockford, IL), and the samples were processed for western blotting. Protein extracts (20 to 60 µg) were boiled for 3 min in Laemmli buffer and separated by 7 to 12% SDS-PAGE (Bio-Rad Laboratories, Hercules, CA), before electrophoretic transfer onto polyvinylidene difluoride membranes. The membranes were blocked in 5% non-fat milk and then probed with an antibody (diluted 1∶1000) that recognized specifically NOS3 (BD Biosciences, BD Transduction Laboratories, San Jose, California), PTEN, phospho-NOS3(S1177), phospho-Akt(S473), phospho-Akt(T308), total Akt (all from Cell Signaling Technology, Danvers, MA), and GAPDH (Abcam, Inc., Cambridge, MA), which served as a loading control. Analysis of published cDNA sequence of rat NOS3 (accession # NM_021838) showed that the serine residue that corresponded to S1177 in the human NOS3 sequence and S1179 in the bovine sequence was at position 1176. In this paper, phosphorylation of this serine residue was therefore referred to as phospho-NOS3(S1176). The membranes were developed in standard fashion (SuperSignal West Dura Chemiluminescent Substrate; Thermo Fisher Scientific Pierce Protein Research Products, Rockford, IL); density of the bands was quantified using Quantity One software (Bio-Rad Laboratories, Hercules, CA).

### Statistical Analyses

Data were expressed as the mean±SEM. Significant differences were determined by two-tailed unpaired t-test or by ANOVA with posthoc testing, as appropriate. Where applicable, general linear models (GLM) were constructed and included age and diet as independent variables. Proc GLM with post-hoc analyses using Tukey’s HSD adjustment were used for each outcome measure (SAS version 9.2, SAS Institute, Inc., Cary, NC). A p-value <0.05 assigned statistical significance.

**Figure 11:**
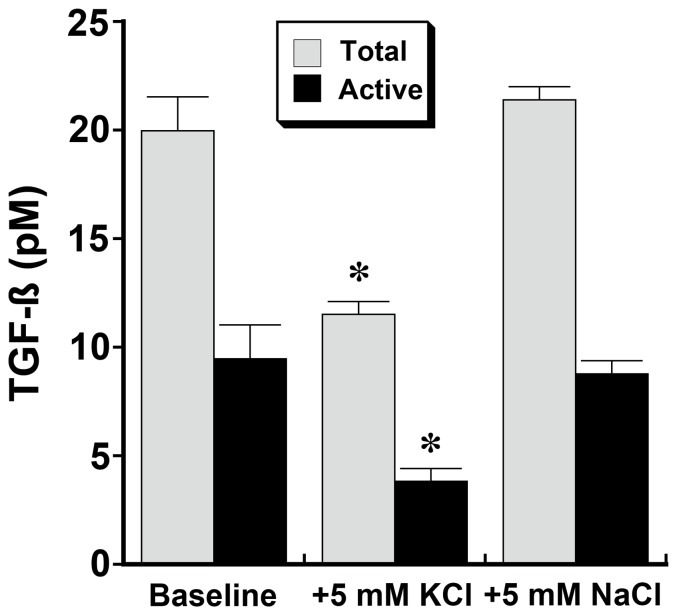
Medium [KCl] affects endothelial cell production of total and active TGF-ß. Medium concentrations of total and active TGF-ß were determined following overnight incubation in medium that contained [KCl], 0 mM, (termed Baseline), and in medium supplemented with 5 mM of either KCl or NaCl. Addition of KCl, but not NaCl, decreased production of both total and active TGF-ß. *p-value <0.05 compared to the other two groups. n = 6 experiments in each group.

**Figure 12:**
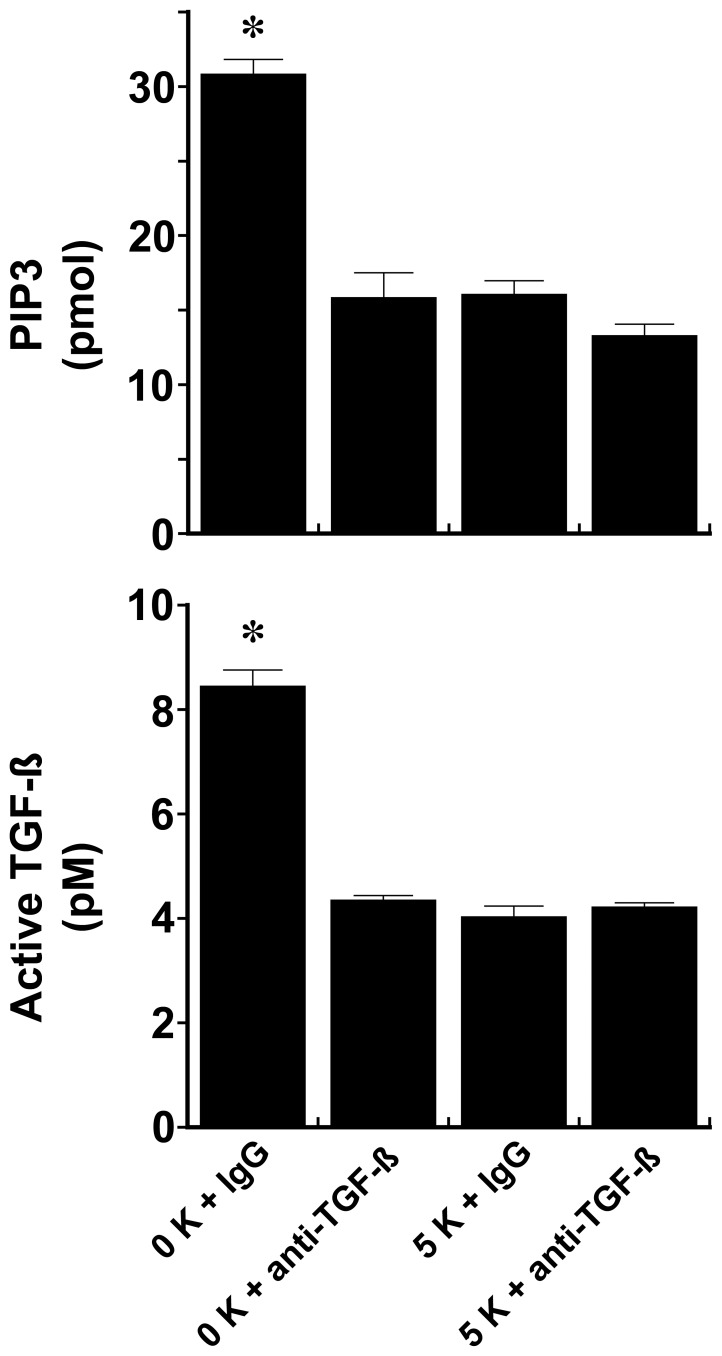
Endothelial cells were incubated overnight in medium that contained [KCl], 0 and 5 mM, and a neutralizing anti-TGF-ß antibody or nonspecific IgG. The observed increase in PIP3 content in cells in the low-potassium medium was inhibited by the addition of the anti-TGF-ß antibody, which did not affect PIP3 levels in cells incubated in medium containing 5 mM [KCl]. Addition of anti-TGF-ß antibody reduced active TGF-ß levels to that observed when medium [KCl] concentration was 5 mM. *p-value <0.05 compared to the other two groups. n = 6 experiments in each group.

**Figure 13:**
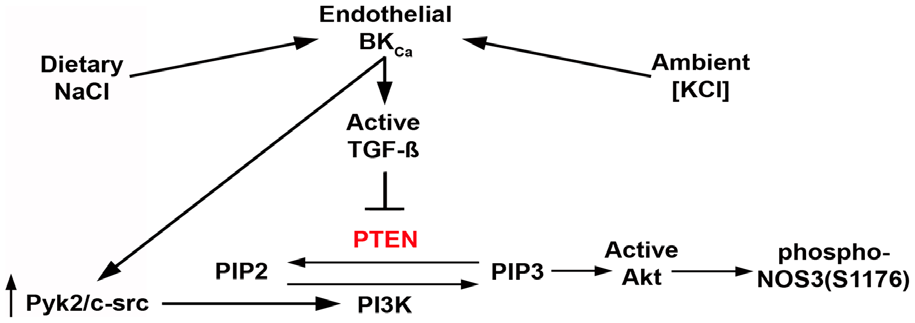
Schematic representation of the findings of the present studies, which focused on PTEN. Prior studies demonstrated that dietary salt intake increased endothelial production of TGF-ß, while potassium mitigated this effect [32], [33], [40], [42], [43], [54]. The published findings further demonstrated an integral role for the endothelial BK_Ca_ channel in this process [40]. The present study demonstrated that endothelial PTEN levels are regulated by factors that include dietary salt intake, which increases TGF-ß, and exogenous TGF-ß. The present study further elucidated in detail the mechanism by which ambient potassium levels regulated PTEN levels and Akt activity through TGF-ß. Endothelial PTEN and phospho-NOS3(S1176) levels responded to changes in dietary salt intake in young animals. In young animals, an increase in TGF-ß is associated with increased NOS3 activity and NO production. With aging, however, PTEN levels increased. By 12 months of age, PTEN levels did not change and phospho-NOS3(S1176) did not increase during increased salt intake. The increase in TGF-ß during aging was not associated with a concomitant increase in NO, which has been shown to mitigate endothelial TGF-ß production [43]. The potential consequence of diminished NO production and unopposed TGF-ß production is altered vascular structure and function, particularly in the setting of high salt intake.

## Results

### Bioavailable NO Production Declined with Age and in Response to Increased Dietary Salt Intake and was Related to Reduced Phospho-NOS3(S1176) and Increased PTEN

Rats were maintained on 0.3% and 8.0% NaCl diets for four days. On the final day of the study, mean body weights (BW), mean arterial pressure (MAP), and serum creatinine concentrations did not differ between the two groups of rats at the same age on the two diets (Table 1). With increasing age, all of these parameters increased. There was a significant linear trend for increasing MAP with age (all p-values<0.05). The MAP least square means for 1-, 6-, and 12-month old rats were 94, 107, and 118 mm Hg, respectively.

As anticipated from the work of Hill, et al. [37], there was a strongly significant inverse linear effect for decreasing urinary NOx with age (p-value<0.0001) (Table 1). Further evaluation demonstrated that NOx values for the 1-month old group (least square mean value of 24 nmol/h per 100 g BW) differed from both the 6- and 12-month old groups (p-value<0.0001). Urinary NOx excretion rates of 6- and 12-month old rats did not differ (8 versus 6 nmol/h per 100 g BW; p-value = 0.79). Urinary NOx excretion rates of the high-salt-treated animals were greater than that observed in the low-salt-treated rats across age groups.

Aortic rings and isolated glomeruli from the high-salt treated animals demonstrated increased NOx production when compared with tissue preparations from the low-salt treated rats at every age studied (Figure 1). The addition of iberiotoxin, a specific BK_Ca_ channel inhibitor, did not reduce NOx production by tissues from the low-salt rats (data not shown) but inhibited salt-induced NOx production. When examined collectively, a progressive decrease in NOx production was observed in both tissues with increasing age of the rat (Figure 2). Because prior studies showed that NOS3 is a substrate for Akt [19] and this Akt-dependent post-translational modification increased NOx in the setting of high-salt intake [23], phosphorylation of NOS3 at S1176 was determined. Compared with data obtained from rats on the 0.3% NaCl diet, the total amount of NOS3 and phosphorylation of NOS3 at S1176 increased in rats at 1 and 6 months of age during high-salt intake, but not at 12 months (Figure 3). These data are also consistent with prior studies showing that shear-induced Akt-dependent phosphorylation of NOS3 is decreased in aortae of older rats [13].

### Dietary Salt-induced Production of TGF-ß Relative to Bioavailable NO Increased with Age

There was no significant effect of age on urinary active TGF-ß excretion rates (Table 1). Urinary active TGF-ß excretion rates of the high-salt-treated animals were greater than that observed in the low-salt-treated rats across age groups.

Production of total and active TGF-ß by aortic rings and isolated glomeruli from rats ages 1, 6, and 12 months was determined while on the two diets. An increase in dietary salt intake increased tissue production of total and active TGF-ß at every age (Figure 4). When examined relative to NOx production, the amount of active TGF-ß produced by aortic rings and isolated glomeruli increased with increasing age of the rat (Figure 5).

### Endothelial PTEN Levels Increase with Age and become Refractory to Changes in Active TGF-ß

To determine the mechanism of reduced NOx production and Akt activity with age, initial studies determined endothelial PTEN levels, which regulate PIP3 levels and Akt activity [26]–[30]. With increasing age, endothelial PTEN levels increased (Figure 6A). In 1-month old rats, endothelial PTEN levels fell while on the high-salt diet (Figure 6B). In contrast, while increased dietary salt intake provoked an increase in the local production of active TGF-ß (Figure 4), endothelial PTEN levels did not change in 12-month old rats (Figure 6C).

### TGF-ß Dose-dependently Decreases Endothelial PTEN and Increases Phospho-Akt(S473)

TGF-ß has been shown to modulate PTEN in mesangial cells [38] and in macrovascular endothelial cells (HUVEC) partly through upregulation of microRNA-21 [39]. Consistent with these findings, primary cultures of HUVEC incubated overnight in medium containing increasing amounts of TGF-ß demonstrated a dose-dependent decrease in PTEN levels and a concomitant increase in phospho-Akt(S473) (Figure 7).

### Ambient [KCl] Dose-dependently Modifies PTEN and Phospho-Akt(S473) Through the BK_Ca_ Channel and TGF-ß

Prior studies showed that the BK_Ca_ channel and dietary potassium content regulated salt-induced production of TGF-ß [33], [40]. To understand endothelial PTEN regulation in greater detail, HUVEC were incubated overnight in medium containing varying concentrations of KCl. Compared to cells exposed to 0 [KCl], cells incubated in 5 mM KCl showed an increase in PTEN and associated reduction in phospho-Akt(S473) (Figure 8). Cells incubated in medium that served as an osmotic control by containing NaCl at 5 mM instead of KCl demonstrated no changes in phospho-Akt(S473) and PTEN. Addition of KCl to the medium at concentrations between 0 and 5 mM produced dose-dependent increases in PTEN and associated reductions in phospho-Akt(S473) (Figure 9). In these experiments, choline chloride was added between 0 and 5 mM, such that the osmolality of the medium did not differ among the groups under study. Addition of iberiotoxin to medium containing [KCl], 5 mM, decreased PTEN and increased phospho-Akt(S473), as well as phospho-Akt(T308), to levels observed in cells incubated in medium containing no KCl (Figure 10). In contrast, addition of glibenclamide to medium containing [KCl], 5 mM, did not decrease PTEN (data not shown).

The effect of extracellular [KCl] on production of TGF-ß was also determined. Compared to production by cells incubated in medium containing no KCl (baseline), the addition of KCl, but not NaCl, at 5 mM, reduced the production of total and active TGF-ß (Figure 11). Addition of iberiotoxin, but not glibenclamide, increased the production of total and active TGF-ß by cells incubated in medium containing 5 mM KCl to that observed by cells in medium containing 0 mM KCl (data not shown). The role of TGF-ß on Akt activation was then determined by overnight incubation of the cells in medium containing a neutralizing antibody to TGF-ß. Incubation of cells in medium containing 0 mM KCl and anti-TGF-ß antibody abrogated TGF-ß activity and reduced levels of cellular PIP3 to that observed in experiments in which the medium contained 5 mM KCl and either anti-TGF-ß antibody or nonspecific IgG (Figure 12).

## Discussion

Endothelial cell function is a critically important determinant of optimum cardiovascular performance through multiple actions that include production of vasoactive compounds and growth factors. TGF-ß and NO in particular are elaborated by endothelium and promote important autocrine effects on the endothelium and paracrine effects on the subjacent smooth muscle. Prior studies demonstrated that TGF-ß drives NO production through NOS3 [41], [42]. A close interrelationship also exists between the endothelial production of TGF-ß and NO, with coordinated expression present in young normotensive rats as well as a countervailing inhibitory effect of NO demonstrated on TGF-ß production [43]. The mechanism by which this coordinated expression develops has been recently elucidated. In young (1 month) rats, a high-salt diet induced the formation of an endothelial cell-signaling complex that contained proline-rich tyrosine kinase 2 (Pyk2), c-Src (also known as pp60*^c-src^*) and class I PI3K (specifically p85 and p110α) [23]. This signaling complex, which was dependent upon endothelial iberiotoxin-sensitive calcium-dependent potassium (BK_Ca_) channel activity, was directly involved in the endothelial cell production of TGF-ß. In addition, PI3K (p85 and p110α) served as the upstream activator of protein kinase B (Akt), which was directly responsible for phosphorylation of the rat endothelial isoform of nitric oxide synthase (NOS3) at S1176 and thereby promoted an increase in NO production in young animals during high-salt intake [23]. Because Akt activation by PIP3 is dependent not only on PI3K but also the lipid phosphatase known as PTEN, which dephosphorylates PIP3 [44], the role of PTEN was examined in the present study, which revealed several novel findings. Endothelial PTEN levels increased during aging, particularly at 12 months, promoting diminished dietary salt-induced NOS3 phosphorylation at S1176, an Akt-dependent effect [23], and reduced NOx production by aortic rings and isolated glomeruli during both low- and hi-salt intake. In contrast, tissue production of TGF-ß relative to NO production did not decline with age, resulting in increased ratios of active TGF-ß to NOx in tissues as the age of the animals increased. The effect of increased salt intake on NOx production by aortic rings and isolated glomeruli was dependent upon endothelial BK_Ca_ channel function at every age studied. PTEN regulates intracellular PIP3 production in macrovascular endothelial cells (HUVEC); PTEN levels were unaltered by ambient [NaCl] but were regulated in a dose-dependent fashion by ambient [KCl]. Finally, consistent with previous studies [33], [40], the effect of potassium on endothelial PTEN and PIP3 levels was mediated through the BK_Ca_ channel specifically by regulating production of TGF-ß. These findings demonstrated a fundamental role for PTEN, which was controlled early in life in part by ambient active TGF-ß concentration, in endothelial cell function (Figure 13).

The importance of balance between PI3K and PTEN has been elucidated. These enzymes critically regulate intracellular PIP3 levels in an interdependent fashion [26]–[29]. PIP3 activates pleckstrin-homology domain-containing enzymes that include Akt, [30] which facilitates NOS3 phosphorylation and NO production [22], and Tec family kinases, which are integrally involved in intracellular calcium signaling [45]. The biological relevance of this interaction in the endothelium has been observed in several conditions. For example, a role for increased NO production through Akt activation has been demonstrated during increased dietary salt intake [23] and in vascular permeability [46] and Flt-1 signaling [47] induced by vascular endothelial growth factor (VEGF). Statins activate endothelial Akt, which results in phosphorylation of NOS3 and enhanced angiogenesis in a model of limb ischemia [48]. The mechanism by which nitroglycerin increases endothelial NO production has recently been shown to be related to inhibition of PTEN, which promotes increases in PIP3 levels, permitting Akt activation and NOS3 phosphorylation [49]. Thus, the PI3K/PTEN pathway may be altered by disease states and by pharmacologic agents. The present study demonstrated the role of aging on this pathway in rats and also fits well with an observed increase in PTEN in cultured microvascular endothelial cells obtained from two aged individuals [50].

In summary, the present findings refine a proposed model of endothelial cell function. A series of in vivo and in vitro studies demonstrated that with aging endothelial PTEN levels increase, thereby promoting a decrease in Akt kinase activity, NOS3 phosphorylation and NO production. This mechanism participates in the age-related impairment in endothelium-dependent vasodilation observed in rats [9], [10]. These effects occurred by “middle age” (12 months), before the development of marked increases in blood pressure and associated reduction in glomerular filtration rate that develops with advanced age (>20 months) in rats [37]. Further, a critical role for TGF-ß in endothelial PTEN regulation was observed. During high salt intake, a signaling complex (Pyk2/c-Src/PI3K) is activated, promoting endothelial production of TGF-ß and NO, the latter through Akt activation [23]. The production of NO is facilitated by TGF-ß-mediated reduction in PTEN, and thus permits completing a negative feedback loop to regulate production of TGF-ß [43], [51], [52]. However, the age-related increase in the “setpoint” relationship between active TGF-ß and NO and the observed resistance to change in endothelial PTEN expression despite an increase in TGF-ß raise the possibility of progressive loss of inhibition of the pathways driven by TGF-ß with aging. This imbalance may be further exacerbated by oxidative stress-mediated reduction in bioavailable NO and enhanced peroxynitrite formation particularly in advanced age [10], [11]. The anticipated consequence of diminished bioavailable NO mediated by both decreased NOS3 phosphorylation and oxidative stress would be arteriosclerosis with increased stiffness in compliance vessels, an important accompaniment of the aging process and cardiovascular risk factor [12], [13], [15], and endothelial cell senescence [53]. Finally, ambient potassium levels modify endothelial TGF-ß production in vitro (Figure 8) and dietary potassium intake functions similarly in vivo and reduces salt-induced production of TGF-ß in young rats [40], raising the potential for improved vascular function through changes in diet.

## References

[1] Furchgott RF, Zawadzki JV (1980) The obligatory role of endothelial cells in the relaxation of arterial smooth muscle by acetylcholine. Nature (Lond) 288: 373–376. 6253831 10.1038/288373a0

[2] Luscher TF, Tanner FC, Tschudi MR, Noll G (1993) Endothelial dysfunction in coronary artery disease. Annu Rev Med 44: 395–418. 8476260 10.1146/annurev.me.44.020193.002143

[3] Goligorsky MS (2005) Endothelial cell dysfunction: can’t live with it, how to live without it. Am J Physiol Renal Physiol 288: F871–880. 15821252 10.1152/ajprenal.00333.2004

[4] Lloyd-Jones D, Adams RJ, Brown TM, Carnethon M, Dai S, et al (2010) Heart disease and stroke statistics–2010 update: a report from the American Heart Association. Circulation 121: e46–e215. 20019324 10.1161/CIRCULATIONAHA.109.192667

[5] Abdu TA, Elhadd T, Pfeifer M, Clayton RN (2001) Endothelial dysfunction in endocrine disease. Trends Endocrinol Metab 12: 257–265. 11445443 10.1016/s1043-2760(01)00425-8

[6] Widlansky ME, Gokce N, Keaney JF Jr, Vita JA (2003) The clinical implications of endothelial dysfunction. J Am Coll Cardiol 42: 1149–1160. 14522472 10.1016/s0735-1097(03)00994-x

[7] Taddei S, Virdis A, Ghiadoni L, Salvetti G, Bernini G, et al (2001) Age-related reduction of NO availability and oxidative stress in humans. Hypertension 38: 274–279. 11509489 10.1161/01.hyp.38.2.274

[8] Celermajer DS, Sorensen KE, Spiegelhalter DJ, Georgakopoulos D, Robinson J, et al (1994) Aging is associated with endothelial dysfunction in healthy men years before the age-related decline in women. J Am Coll Cardiol 24: 471–476. 8034885 10.1016/0735-1097(94)90305-0

[9] Hongo K, Nakagomi T, Kassell NF, Sasaki T, Lehman M, et al (1988) Effects of aging and hypertension on endothelium-dependent vascular relaxation in rat carotid artery. Stroke 19: 892–897. 3388460 10.1161/01.str.19.7.892

[10] van der Loo B, Labugger R, Skepper JN, Bachschmid M, Kilo J, et al (2000) Enhanced peroxynitrite formation is associated with vascular aging. J Exp Med 192: 1731–1744. 11120770 10.1084/jem.192.12.1731 PMC2213492

[11] Blackwell KA, Sorenson JP, Richardson DM, Smith LA, Suda O, et al (2004) Mechanisms of aging-induced impairment of endothelium-dependent relaxation: role of tetrahydrobiopterin. Am J Physiol Heart Circ Physiol 287: H2448–2453. 15319209 10.1152/ajpheart.00248.2004

[12] Lakatta EG, Levy D (2003) Arterial and cardiac aging: major shareholders in cardiovascular disease enterprises: Part I: aging arteries: a “set up” for vascular disease. Circulation 107: 139–146. 12515756 10.1161/01.cir.0000048892.83521.58

[13] Soucy KG, Ryoo S, Benjo A, Lim HK, Gupta G, et al (2006) Impaired shear stress-induced nitric oxide production through decreased NOS phosphorylation contributes to age-related vascular stiffness. J Appl Physiol 101: 1751–1759. 17106067 10.1152/japplphysiol.00138.2006

[14] Halcox JP, Donald AE, Ellins E, Witte DR, Shipley MJ, et al (2009) Endothelial function predicts progression of carotid intima-media thickness. Circulation 119: 1005–1012. 19204308 10.1161/CIRCULATIONAHA.108.765701

[15] O’Leary DH, Polak JF, Kronmal RA, Manolio TA, Burke GL, et al (1999) Carotid-artery intima and media thickness as a risk factor for myocardial infarction and stroke in older adults. Cardiovascular Health Study Collaborative Research Group. N Engl J Med 340: 14–22. 9878640 10.1056/NEJM199901073400103

[16] Laurent S, Boutouyrie P (2007) Recent advances in arterial stiffness and wave reflection in human hypertension. Hypertension 49: 1202–1206. 17452508 10.1161/HYPERTENSIONAHA.106.076166

[17] O’Rourke MF, Safar ME (2005) Relationship between aortic stiffening and microvascular disease in brain and kidney: cause and logic of therapy. Hypertension 46: 200–204. 15911742 10.1161/01.HYP.0000168052.00426.65

[18] Cohen DL, Townsend RR (2011) Central blood pressure and chronic kidney disease progression. Int J Nephrol 2011: 407801. 21423561 10.4061/2011/407801 PMC3056344

[19] Fulton D, Gratton JP, McCabe TJ, Fontana J, Fujio Y, et al (1999) Regulation of endothelium-derived nitric oxide production by the protein kinase Akt. Nature 399: 597–601. 10376602 10.1038/21218 PMC3637917

[20] Fulton D, Gratton JP, Sessa WC (2001) Post-translational control of endothelial nitric oxide synthase: why isn’t calcium/calmodulin enough? J Pharmacol Exp Ther 299: 818–824. 11714864

[21] Bauer PM, Fulton D, Boo YC, Sorescu GP, Kemp BE, et al (2003) Compensatory phosphorylation and protein-protein interactions revealed by loss of function and gain of function mutants of multiple serine phosphorylation sites in endothelial nitric-oxide synthase. J Biol Chem 278: 14841–14849. 12591925 10.1074/jbc.M211926200

[22] Lane P, Gross SS (2002) Disabling a C-terminal autoinhibitory control element in endothelial nitric-oxide synthase by phosphorylation provides a molecular explanation for activation of vascular NO synthesis by diverse physiological stimuli. J Biol Chem 277: 19087–19094. 11839759 10.1074/jbc.M200258200

[23] Ying WZ, Aaron K, Sanders PW (2008) Dietary salt activates an endothelial proline-rich tyrosine kinase 2/c-Src/phosphatidylinositol 3-kinase complex to promote endothelial nitric oxide synthase phosphorylation. Hypertension 52: 1134–1141. 18981321 10.1161/HYPERTENSIONAHA.108.121582 PMC2680421

[24] Li J, Yen C, Liaw D, Podsypanina K, Bose S, et al (1997) PTEN, a putative protein tyrosine phosphatase gene mutated in human brain, breast, and prostate cancer. Science 275: 1943–1947. 9072974 10.1126/science.275.5308.1943

[25] Maehama T, Dixon JE (1998) The tumor suppressor, PTEN/MMAC1, dephosphorylates the lipid second messenger, phosphatidylinositol 3,4,5-trisphosphate. J Biol Chem 273: 13375–13378. 9593664 10.1074/jbc.273.22.13375

[26] Vanhaesebroeck B, Leevers SJ, Ahmadi K, Timms J, Katso R, et al (2001) Synthesis and function of 3-phosphorylated inositol lipids. Annu Rev Biochem 70: 535–602. 11395417 10.1146/annurev.biochem.70.1.535

[27] Vanhaesebroeck B, Stephens L, Hawkins P (2012) PI3K signalling: the path to discovery and understanding. Nat Rev Mol Cell Biol 13: 195–203. 22358332 10.1038/nrm3290

[28] Stambolic V, Suzuki A, de la Pompa JL, Brothers GM, Mirtsos C, et al (1998) Negative regulation of PKB/Akt-dependent cell survival by the tumor suppressor PTEN. Cell 95: 29–39. 9778245 10.1016/s0092-8674(00)81780-8

[29] Mellor P, Furber LA, Nyarko JN, Anderson DH (2012) Multiple roles for the p85alpha isoform in the regulation and function of PI3K signalling and receptor trafficking. Biochem J 441: 23–37. 22168437 10.1042/BJ20111164

[30] Stocker H, Andjelkovic M, Oldham S, Laffargue M, Wymann MP, et al (2002) Living with lethal PIP3 levels: viability of flies lacking PTEN restored by a PH domain mutation in Akt/PKB. Science 295: 2088–2091. 11872800 10.1126/science.1068094

[31] Takahashi N, Boysen G, Li F, Li Y, Swenberg JA (2007) Tandem mass spectrometry measurements of creatinine in mouse plasma and urine for determining glomerular filtration rate. Kidney Int 71: 266–271. 17149371 10.1038/sj.ki.5002033

[32] Ying WZ, Aaron K, Sanders PW (2008) Mechanism of dietary salt-mediated increase in intravascular production of TGF-beta1. Am J Physiol Renal Physiol 295: F406–414. 18562633 10.1152/ajprenal.90294.2008 PMC2519184

[33] Ying WZ, Sanders PW (1998) Dietary salt modulates renal production of transforming growth factor-beta in rats. Am J Physiol 274: F635–641. 9575885 10.1152/ajprenal.1998.274.4.F635

[34] Galvez A, Gimenez-Gallego G, Reuben JP, Roy-Contancin L, Feigenbaum P, et al (1990) Purification and characterization of a unique, potent, peptidyl probe for the high conductance calcium-activated potassium channel from venom of the scorpion Buthus tamulus. J Biol Chem 265: 11083–11090. 1694175

[35] Sturgess NC, Ashford ML, Cook DL, Hales CN (1985) The sulphonylurea receptor may be an ATP-sensitive potassium channel. Lancet 2: 474–475. 2412077 10.1016/s0140-6736(85)90403-9

[36] Gray A, Olsson H, Batty IH, Priganica L, Peter Downes C (2003) Nonradioactive methods for the assay of phosphoinositide 3-kinases and phosphoinositide phosphatases and selective detection of signaling lipids in cell and tissue extracts. Anal Biochem 313: 234–245. 12605860 10.1016/s0003-2697(02)00607-3

[37] Hill C, Lateef AM, Engels K, Samsell L, Baylis C (1997) Basal and stimulated nitric oxide in control of kidney function in the aging rat. Am J Physiol 272: R1747–1753. 9227586 10.1152/ajpregu.1997.272.6.R1747 PMC2756800

[38] Kato M, Putta S, Wang M, Yuan H, Lanting L, et al (2009) TGF-beta activates Akt kinase through a microRNA-dependent amplifying circuit targeting PTEN. Nat Cell Biol 11: 881–889. 19543271 10.1038/ncb1897 PMC2744130

[39] Kumarswamy R, Volkmann I, Jazbutyte V, Dangwal S, Park DH, et al (2012) Transforming growth factor-beta-induced endothelial-to-mesenchymal transition is partly mediated by microRNA-21. Arterioscler Thromb Vasc Biol 32: 361–369. 22095988 10.1161/ATVBAHA.111.234286

[40] Ying WZ, Aaron K, Wang PX, Sanders PW (2009) Potassium inhibits dietary salt-induced transforming growth factor-beta production. Hypertension 54: 1159–1163. 19738156 10.1161/HYPERTENSIONAHA.109.138255 PMC2766016

[41] Ying WZ, Sanders PW (1998) Dietary salt enhances glomerular endothelial nitric oxide synthase through TGF-beta1. Am J Physiol 275: F18–24. 9689000 10.1152/ajprenal.1998.275.1.F18

[42] Ying WZ, Sanders PW (1999) Dietary salt increases endothelial nitric oxide synthase and TGF-beta1 in rat aortic endothelium. Am J Physiol 277: H1293–1298. 10516163 10.1152/ajpheart.1999.277.4.H1293

[43] Ying WZ, Sanders PW (2003) The interrelationship between TGF-beta1 and nitric oxide is altered in salt-sensitive hypertension. Am J Physiol Renal Physiol 285: F902–908. 12865256 10.1152/ajprenal.00177.2003

[44] Jiang BH, Liu LZ (2009) PI3K/PTEN signaling in angiogenesis and tumorigenesis. Adv Cancer Res 102: 19–65. 19595306 10.1016/S0065-230X(09)02002-8 PMC2933405

[45] Scharenberg AM, Kinet JP (1998) PtdIns-3,4,5-P3: a regulatory nexus between tyrosine kinases and sustained calcium signals. Cell 94: 5–8. 9674420 10.1016/s0092-8674(00)81214-3

[46] Six I, Kureishi Y, Luo Z, Walsh K (2002) Akt signaling mediates VEGF/VPF vascular permeability in vivo. FEBS Lett 532: 67–69. 12459464 10.1016/s0014-5793(02)03630-x

[47] LeBlanc AJ, Shipley RD, Kang LS, Muller-Delp JM (2008) Age impairs Flk-1 signaling and NO-mediated vasodilation in coronary arterioles. Am J Physiol Heart Circ Physiol 295: H2280–2288. 18835919 10.1152/ajpheart.00541.2008 PMC2614537

[48] Kureishi Y, Luo Z, Shiojima I, Bialik A, Fulton D, et al (2000) The HMG-CoA reductase inhibitor simvastatin activates the protein kinase Akt and promotes angiogenesis in normocholesterolemic animals. Nat Med 6: 1004–1010. 10973320 10.1038/79510 PMC2828689

[49] Mao M, Sudhahar V, Ansenberger-Fricano K, Fernandes DC, Tanaka LY, et al (2012) Nitroglycerin drives endothelial nitric oxide synthase activation via the phosphatidylinositol 3-kinase/protein kinase B pathway. Free Radic Biol Med 52: 427–435. 22037515 10.1016/j.freeradbiomed.2011.09.020 PMC3432314

[50] Tarnawski AS, Pai R, Tanigawa T, Matysiak-Budnik T, Ahluwalia A (2010) PTEN silencing reverses aging-related impairment of angiogenesis in microvascular endothelial cells. Biochem Biophys Res Commun 394: 291–296. 20193662 10.1016/j.bbrc.2010.02.161

[51] Saura M, Zaragoza C, Herranz B, Griera M, Diez-Marques L, et al (2005) Nitric oxide regulates transforming growth factor-beta signaling in endothelial cells. Circ Res 97: 1115–1123. 16239590 10.1161/01.RES.0000191538.76771.66

[52] Wang S, Shiva S, Poczatek MH, Darley-Usmar V, Murphy-Ullrich JE (2002) Nitric oxide and cGMP-dependent protein kinase regulation of glucose-mediated thrombospondin 1-dependent transforming growth factor-ß activation in mesangial cells. J Biol Chem 277: 9880–9888. 11784717 10.1074/jbc.M108360200

[53] Hayashi T, Matsui-Hirai H, Miyazaki-Akita A, Fukatsu A, Funami J, et al (2006) Endothelial cellular senescence is inhibited by nitric oxide: implications in atherosclerosis associated with menopause and diabetes. Proc Natl Acad Sci U S A 103: 17018–17023. 17075048 10.1073/pnas.0607873103 PMC1629003

[54] Ying W-Z, Sanders PW (2002) Increased dietary salt activates rat aortic endothelium. Hypertension 39: 239–244. 11847191 10.1161/hy0202.104142

